# Simultaneous profiling and quantification of 25 eicosanoids in human serum by ultrahigh-performance liquid chromatography coupled to tandem mass spectrometry

**DOI:** 10.1007/s00216-022-04351-6

**Published:** 2022-11-08

**Authors:** Yuanyuan Lu, Zhitong Mai, Hongxia Zhou, Wenda Guan, Shiguan Wu, Heyan Zou, Maoting Shen, Yangqing Zhan, Feng Ye, Minshan Qiu, Lihan Shen, Beibei Zhao, Zifeng Yang

**Affiliations:** 1 State Key Laboratory of Respiratory Disease, National Clinical Research Center for Respiratory Disease, Guangzhou Institute of Respiratory Health, the First Affiliated Hospital of Guangzhou Medical University, Guangdong 510120 Guangzhou, People’s Republic of China; 2 Guangzhou KingMed Center for Clinical Laboratory Co.Ltd, Guangdong 510000 Gaungzhou, People’s Republic of China; 3 Department of Critical Care Medicine, Dongguan Institute of Respiratory and Critical Care Medicine, Affiliated Dongguan Hospital, Southern Medical University, Guangdong 523059 Dongguan, People’s Republic of China; 4 State Key Laboratory of Quality Research in Chinese Medicine, Macau Institute for Applied Research in Medicine and Health, Macau University of Science and Technology, Macau SAR Taipa, People’s Republic of China; 5 Guangzhou Laboratory, 510000 Guangzhou, People’s Republic of China; 6 Guangzhou Key Laboratory for Clinical Rapid Diagnosis and Early Warning of Infectious Diseases, Guangdong 510000 Guangzhou, People’s Republic of China

**Keywords:** Eicosanoids, LC–MS/MS, H1N1, Bacteria, Severe pneumonia

## Abstract

The eicosanoid metabolic pathway is responsible for mediating the production of various inflammatory factors that are closely related to the development and resolution of inflammation. In biological matrices, the major quantifying obstacles were shown to be the oxidation and low quantities of eicosanoids and their metabolites. This study aimed to develop a reliable, sensitive ultrahigh-performance liquid chromatography coupled to a tandem mass spectrometry (UPLC–MS/MS) method to quantify eicosanoids in human serum. Solid-phase extraction (SPE) was used for sample preparation. The approach employed continuous ionization polarity switching. The target eicosanoids showed good linearity over the investigated concentration range (*r*2 > 0.99). The recovery rates were over 64.5%, and the matrix effects ranged from 73.0 to 128.0%. The limits of quantification were 0.048 ~ 0.44 ng/mL. For the broad concentration range, the CV % for accuracy and precision were less than ± 20%. We successfully applied this method to rapidly analyse 74 serum samples from severe influenza pneumonia, severe bacterial pneumonia and healthy individuals. Eicosanoid-related metabolite concentrations were quantified within a range similar to those of previously published articles. Compared to healthy individuals, our application found that 20-HETE, 14,15-EET and 11,12-EET were upregulated in severe influenza pneumonia patients, while LTB_4_ was downregulated. 8-HETE and 5-HETE were upregulated in severe bacterial pneumonia patients, while LTE_4_ was downregulated. This approach provides a means for monitoring the low quantities of eicosanoids in biological matrices, and our finding that different characteristic metabolite profiles may help discriminate the induction of severe pneumonia patients.

**Supplementary Information:**

The online version contains supplementary material available at 10.1007/s00216-022-04351-6.

## Introduction

Microbial infection and inflammation are accompanied by cytokine-induced alterations in lipid metabolism. Lipids play crucial roles at various stages in the virus life cycle. Furthermore, lipids are an integral part of the innate and adaptive immune system.

Several studies have indicated that the process of inflammation is widely regulated by lipids and their mediators during influenza virus infection [1–3]. In particular, the arachidonic acid (AA) pathway plays an important role in physiological processes and pathophysiological conditions [4]. AA belongs to a subclass of unsaturated fatty acids of the fatty acyl family, which is liberated from membrane phospholipids by PLA2. AA is metabolized into many potent bioactive eicosanoids by cyclooxygenase (COX), cytochrome P450 (CYP450) and lipoxygenase (LOX). Prostaglandins (PGs) and thromboxanes (TXs) are COX products. Hydroxyeicosatetraenoic acids (HETEs) and leukotrienes (LTs) are mainly derived from LOX. Epoxy-eicosatrienoic acids (EETs) and several HETEs are products of CYP450. Eicosanoids act as pro- and anti-inflammatory regulators. H5N1 infection induced extensive COX-2 production in epithelial cells that triggered a proinflammatory cascade in the lung tissue of patients with fatal outcomes [5]. An integrated omics analysis of pathogenic host responses during pandemic H1N1 influenza virus infection indicated that the proinflammatory lipid alteration correlated with severe tracheal lesions after influenza virus infection [6]. Several phospholipids, which can be cleaved to form arachidonic acid (AA), were found to have significant abundance changes. The increase in these phospholipids enhanced inflammatory responses, resulting in tissue damage.

The profile of eicosanoids could be particularly helpful not only in monitoring the course of disease but also in the development of new treatment strategies. Recently, liquid chromatography–tandem mass spectrometry (LC–MS/MS) has been considered to be a superior quantitation technique for eicosanoid profiling because of its high selectivity, sensitivity and accuracy [7, 8]. However, the development of these techniques has been challenging due to structural similarity and variance in endogenous concentrations. To avoid signal interference between the studied eicosanoids, the specificity of the LC–MS/MS method should be additionally evaluated because those isomeric compounds can generate not only the same precursor ions but also the same product ions.

The aim of this study was to develop a UPLC–MS/MS method for the simultaneous determination of AA metabolites in human serum samples. Chromatographic separation is paramount when monitoring numerous structurally similar metabolites. We focused on adequate chromatographic separation and multiple reaction monitoring (MRM) mode to ensure detection specificity. The developed method can be used to monitor the changes and different situations of eicosanoids and their metabolites in serum samples during infection. Based on the different levels of metabolites, this research might be helpful for understanding the complex regulatory AA metabolites in response to different infections.

## Materials and methods

### Chemicals and materials

5-Hydroxyeicosatetraenoic acid (5-HETE), 8-hydroxyeicosatetraenoic acid (8-HETE), 9-hydroxyeicosatetraenoic acid (9-HETE), 12-hydroxyeicosatetraenoic acid (12-HETE), 15-hydroxyeicosatetraenoic acid (15-HETE), 20-hydroxyeicosatetraenoic acid (20-HETE), leukotriene B_4_ (LTB_4_), 20-carboxy leukotriene B_4_ (20-COOH-LTB_4_), 20-hydroxy leukotriene B4 (20-OH-LTB_4_), leukotriene C_4_ (LTC_4_), leukotriene D_4_ (LTD_4_), leukotriene E_4_ (LTE_4_), prostaglandin D_2_ (PGD_2_), prostaglandin E_2_ (PGE_2_), prostaglandin H_2_ (PGH_2_), prostaglandin J_2_ (PGJ_2_), Δ^12^-prostaglandin J_2_ (Δ^12^-PGJ_2_), 15-deoxy-Δ^12,14^-prostaglandin J_2_ (15-d-Δ^12,14^-PGJ_2_), prostaglandin A_2_ (PGA_2_), prostaglandin B_2_ (PGB_2_), thromboxane B_2_ (TXB_2_), 5-oxo-eicosatrienoic acid (5-oxo-EET), 8-iso prostaglandin F_2α_(8-iso PGF_2α_), ( ±)5,6-epoxy-eicosatrienoic acid (( ±)5,6-EET), ( ±)8,9-epoxy-eicosatrienoic acid (( ±)8,9-EET), ( ±)11,12-epoxy-eicosatrienoic acid (( ±)11,12-EET) and ( ±)14,15-epoxy-eicosatrienoic acid (( ±)14,15-EET) were purchased from Cayman Chemicals (Ann Arbor, MI, USA). Deuterated internal standards PGE_2_-d4, TXB_2_-d4, PGD_2_-d4, 8-iso-PGF2α-d4, PGA_2_-d4, LTE_4_-d5, LTC_4_-d5, LTB_4_-d4, 5-HETE-d8, 12-HETE-d8, 15-HETE-d8, 20-HETE-d6, ( ±)14,15-EET-d11, ( ±)5,6-EET-d11 and ( ±) 8,9-EET-d11 were also obtained from Cayman Chemicals.

HPLC-grade solvents and GC-grade 2,6-di-tert-butyl-4-methylphenol (BHT) were purchased from Sigma Aldrich (St Louis, MO, USA). Waters solid-phase extraction (SPE) 96-well plates (Oasis HLB, 5 mg/96-well) were purchased from Waters Co. (Milford, MA, USA). Human serum from defibrinated double charcoal-stripped plasma was obtained from Golden West Diagnostics, LLC (Temecula, CA, USA). All other chemicals and solvents were of the highest analytical grade available.

### Preparation of standard solutions and quality control (QC) samples

For the preparation of calibration curves, stock solutions were prepared in ethanol [containing 0.05% (w/v) BHT] that contained all eicosanoid standards at a concentration of 1 μg/mL for 8-HETE, 9-HETE, 20-HETE, LTB_4_, 20-COOH-LTB_4_, 20-OH-LTB_4_, LTC_4_, LTD_4_, LTE_4_, PGD_2_, PGE_2_, PGH_2_, PGJ_2_, Δ^12^-PGJ_2_, 15-d-Δ^12,14^-PGJ_2_, PGA_2_, PGB_2_, 5-oxo-EET, 8-iso PGF_2α_, ( ±)5,6-EET, ( ±)8,9-EET, ( ±)11,12-EET and ( ±)14,15-EET; 5 μg/mL for TXB_2_ and 15-HETE; and 9 μg/mL for 5-HETE and 12-HETE. Working standard solutions for all eicosanoids were prepared by serial dilution of the stock solutions with 60% (v/v) ethanol [containing 0.05% (w/v) BHT and 0.1% (v/v) formic acid] to create the necessary concentrations. Standard solutions were prepared in blank plasma by spiking with an appropriate volume of the serially diluted stock solutions, resulting in seven different concentrations required for the calibration curve. A solution containing 15 internal (deuterated) eicosanoid standards was prepared in ethanol [containing 0.05% (w/v) BHT] at a concentration of 20 ng/mL for PGE_2_-d4, PGD_2_-d4, 8-iso-PGF_2α_-d4, PGA_2_-d4, LTE_4_-d5, LTC_4_-d5, LTB4-d4, 15-HETE-d8, 20-HETE-d6, ( ±)14,15-EET-d11, ( ±)5,6-EET-d11 and ( ±) 8,9-EET-d11; 200 ng/μL for TXB_2_-d4; and 500 ng/mL for 5-HETE-d8 and 12-HETE-d8. All solutions were stored at − 80 °C when not in use. QC samples were prepared by spiking blank serum with three levels of eicosanoids, including low, medium and high concentrations.

### Sample preparation

Prior to extraction, Waters Oasis-HLB 96-well plates were washed with methanol (0.5 mL) and 0.1% (v/v) formic acid in water (0.5 mL). Two-hundred-microlitre serum samples were spiked with 20 μL of IS mixture followed by diluting with 5% (v/v) formic acid. The mixture was applied to the plate and subsequently washed with 0.5 mL of 5% (v/v) methanol solution [containing 0.1% (v/v) formic acid]. All analytes were eluted with 0.2 mL of methanol [containing 0.05% (w/v) BHT and 0.1% (v/v) formic acid] 2 times. The eluate was evaporated to dryness under a nitrogen stream. The resulting residues were dissolved in 80 μL of methanol/acetonitrile/water [3/3/4, v/v/v, containing 0.05% (w/v) BHT and 0.1% (v/v) formic acid]. The prepared samples were kept in an autosampler at 4 °C and injected into the UPLC–MS/MS system.

### Chromatographic conditions

UPLC was conducted using an Agilent 1290 Infinity series HPLC system. Chromatographic separations were performed on an Agilent InfinityLab Poroshell 120 Phenyl-Hexyl column (2.7 μm, 3.0 cm*150 mm, Agilent, Palo Alto, CA, USA). The column was maintained at 35 °C, and the injection volume was set to 10 μL. Solvent A was 0.05% acetic acid in water, and solvent B consisted of acetonitrile:methanol (1:1, v/v). The mobile phase flow rate was 0.6 mL/min. The gradient was as follows: 0–12.0 min, 45% B; 12.0–12.5 min, 45 to 65% B; 12.5–21.0 min, 65% B; 21.0–21.1 min, 65 to 98% B; 21.1–23.0 min, 98% B; and 23.0–23.1 min, 98 to 45% B and maintained for 2.9 min.

### MS conditions

An Agilent 6495 triple-quadrupole mass spectrometer (Agilent Technologies, Inc. Ltd., CA, USA) with an AJS electrospray ionization (AJS-ESI) was utilized. ESI was performed in negative ionization mode using N_2_ at a pressure of 45 psi for the nebulizer with a flow of 14 L/min and a temperature of 200 °C. The sheath gas temperature was 350 °C with a flow rate of 12 L/min. The capillary voltage was set at 3500 V, and the nozzle voltage was 1500 V. Eicosanoids were analysed using dynamic multiple reaction monitoring (dMRM) mode with negative/positive polarity switching. Mass spectrometric parameters were optimized for each analyte.

### Validation of the method

The developed method was validated according to the US-FDA Bioanalytical Method Validation Guidance(2018) [9].

#### Matrix effect and recovery rate

The absolute matrix effect (%ME_A_) was evaluated by comparison of the detector response for QC samples with the addition of eicosanoids at two levels (low concentration and high concentration) after extraction of the matrix and prepared as neat solutions. The absolute matrix effect was determined by measuring six independent replicates of QC samples per level. The normalized absolute matrix effect was calculated by dividing the absolute matrix effect of the analyte by the absolute matrix effect of the selected IS.

The recovery of analytes (%RE) was assessed by comparison of the detector response for QC samples with the addition of eicosanoids at low and high QC concentrations before and after extraction of the matrix. The ratio between the analyte peak area and internal standard peak area was considered to represent the detector response. The recovery was evaluated based on measurements of six independent replicates of QC samples per level.

#### Calibration curves and the lower limit of quantitation (LLOQ)

Calibration curves were obtained using seven calibration standards and were fitted by weighed (1/*x*) least-squares linear regressions of the response ratios (peak area analyte/peak area internal standard). For each analyte, two calibration curves were performed each day on three different days. The acceptance criteria for each back-calculated concentration of the calibration standards were within ± 15% of the nominal value, except for the LLOQ, for which it was within ± 20%. The LLOQ was defined as a signal-to-noise (*S*/*N*) ratio greater than 10:1.

### Precision and accuracy

The precision and accuracy of the assay were assessed by analysing QC samples under three different concentrations in six replicates on the same day and on three consecutive days for within-run and between-run precision and accuracy, respectively. Precision was expressed as the percent coefficient of variance (CV%), and accuracy was determined from the percentage ratio of the measured concentration to the nominal concentration and represented by the relative error (RE%). The within-run and between-run precision and accuracy should not exceed ± 15%.

### Stability

Stability was assessed by analysing QC samples in triplicate at three levels after three different manipulations: (1) short-term storage of plasma samples (8 h at room temperature), (2) posttreatment storage in the autosampler (8, 14 and 24 h at 4 °C) and (3) three freeze–thaw cycles (− 80 °C to room temperature). Room temperature was defined as 25 °C.

### Research participants and serum sample collection

A total of 74 participants were involved. Among them, 12 patients were infected with H1N1 and diagnosed with severe pneumonia, 28 patients were infected with bacteria and diagnosed with severe pneumonia and the other individuals were defined as healthy controls. The patients were all recruited from the intensive care unit (ICU) of the hospital. The study was approved by the ethical committee of the First Affiliated Hospital of Guangzhou Medical University (Ethics No. 2016-78) and the ethical committee of the Affiliated Dongguan Hospital, Southern Medical University (Ethics No. KYKT2021-031). Briefly, serum samples (*n* = 74) were collected from individuals at the First Affiliated Hospital of Guangzhou Medical University from March to October 2019 and the Affiliated Dongguan Hospital, Southern Medical University, in June 2021. Then, blood was collected in pro-coagulation tubes and clotted at room temperature for 30 min. Samples were centrifuged at 3000 rpm for 10 min to separate the serum. Finally, the serum samples were stored in a − 80 °C freezer until analysis.

### Data processing and statistical analysis

Data acquisition and processing were performed using Agilent MassHunter Quantitative Analysis software Version 10.1. SIMCA version 15.0.2 (Sartorious Stedim Biotech, Umea, Sweden) was used for statistical data analysis. SPSS was used for Student’s *t* test to compare data between two groups; *P* < 0.05 was considered statistically significant.

## Results and discussion

### Optimization of the LC–MS/MS conditions

**Fig. 1:**
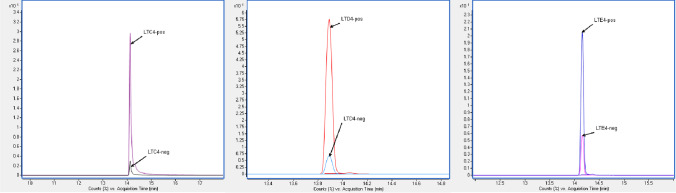
Chromatograms for LTC4 (left), LTD4 (middle) and LTE4 (right). Overlaid dMRM traces for the positive and negative ion modes are shown

MRM conditions were selected from the spectra obtained by direct injection of the individual standard solutions into the mass spectrometer. Most of the compounds were ionized dominantly or only in negative ionization mode. However, 5 compounds, including 2 internal standards (LTC_4_, LTD_4_, LTE_4_, LTE_4_-d5 and LTC_4_-d5), were better ionized in positive ionization mode. The signal intensities were two- to ninefold higher in positive ionization mode than in negative ionization mode, which was consistent with the results of previous work [10]. Consequently, a polarity switching method was conducted for eicosanoid profiling in one run. The chromatograms of LTC_4_, LTD_4_ and LTE_4_ obtained by continuous polarity switching are shown in Fig. 1.

To improve sensitivity and peak symmetry, we employed a dMRM procedure. dMRM utilizes a constant cycle time to ensure a uniform distribution of data points [11], which will improve the chromatographic peaks with better peak symmetry that enables reproducibility in retention time, peak areas and accuracy of quantitation. The optimal parameters for target compounds, including collision energy (CE) and transitions, are shown in Table 1.

**Table 1:** Dynamic multiple reaction monitoring (dMRM) parameters for eicosanoids and internal (deuterated) standards

Name	Transition (*m/z*)	CE (V)	Polarity
20-COOH-LTB_4_	365.2 > 347.1	16	Negative
20-OH-LTB_4_	351.2 > 195.0	12	Negative
8-i-PGF_2a_	353.2 > 193.0	24	Negative
TXB_2_	369.2 > 169.1	16	Negative
PGE_2_	351.2 > 271.1	12	Negative
PGD_2_	351.2 > 271.0	12	Negative
PGH_2_	351.2 > 271.2	16	Negative
PGA_2_	333.2 > 270.9	12	Negative
12-PGJ_2_	333.2 > 271.1	8	Negative
PGB_2_	333.2 > 235.1	20	Negative
PGJ_2_	333.2 > 232.9	4	Negative
LTB_4_	335.2 > 195.1	12	Negative
LTC_4_	626.3 > 189.1	20	Positive
LTE_4_	440.2 > 189.2	8	Positive
15D-PGJ_2_	315.2 > 270.9	12	Negative
LTD_4_	497.2 > 189.1	12	Positive
20-HETE	319.2 > 289.1	16	Negative
15-HETE	319.2 > 219.0	8	Negative
12-HETE	319.2 > 179.0	16	Negative
8-HETE	319.2 > 155.1	12	Negative
9-HETE	319.2 > 151.1	12	Negative
14,15-EET	319.2 > 218.9	8	Negative
5-HETE	319.2 > 114.9	12	Negative
11,12-EET	319.2 > 167.1	12	Negative
5-OXO-EET	317.2 > 203.2	16	Negative
8,9-EET	319.2 > 155.0	12	Negative
5,6-EET	319.2 > 191.1	8	Negative
8-i-PGF_2a_-d4	357.3 > 197.0	28	Negative
TXB_2_-d4	373.2 > 173.0	12	Negative
PGE_2_-d4	355.2 > 275.1	20	Negative
PGD_2_-d4	355.2 > 275.1	16	Negative
PGA_2_-d4	337.2 > 275.1	16	Negative
LTC_4_-d5	631.3 > 194.1	20	Positive
LTB_4_-d4	339.2 > 197.1	12	Negative
LTE_4_-d5	445.3 > 306.1	8	Positive
20-HETE-d6	325.3 > 281.2	16	Negative
12-HETE-d8	327.3 > 184.1	12	Negative
5-HETE-d8	327.3 > 116.1	16	Negative
14,15-EET-d11	330.3 > 268.2	8	Negative
11,12-EET-d11	330.3 > 167.0	12	Negative
8,9-EET-d11	330.3 > 268.3	12	Negative
5,6-EET-d11	330.3 > 202.1	12	Negative

**Fig. 2:**
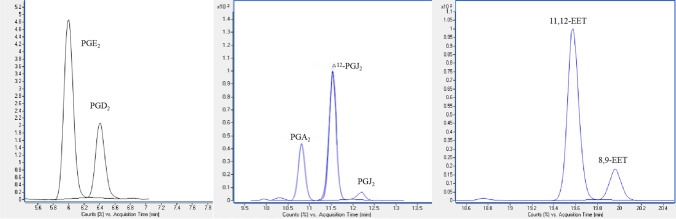
Chromatograph of critical separation: PGD2/PGE2 (left), PGA_2_/ PGJ_2_/**△**^12^-PGJ_2_ (middle) and 8,9-EET/11,12-EET (right)

**Fig. 3:**
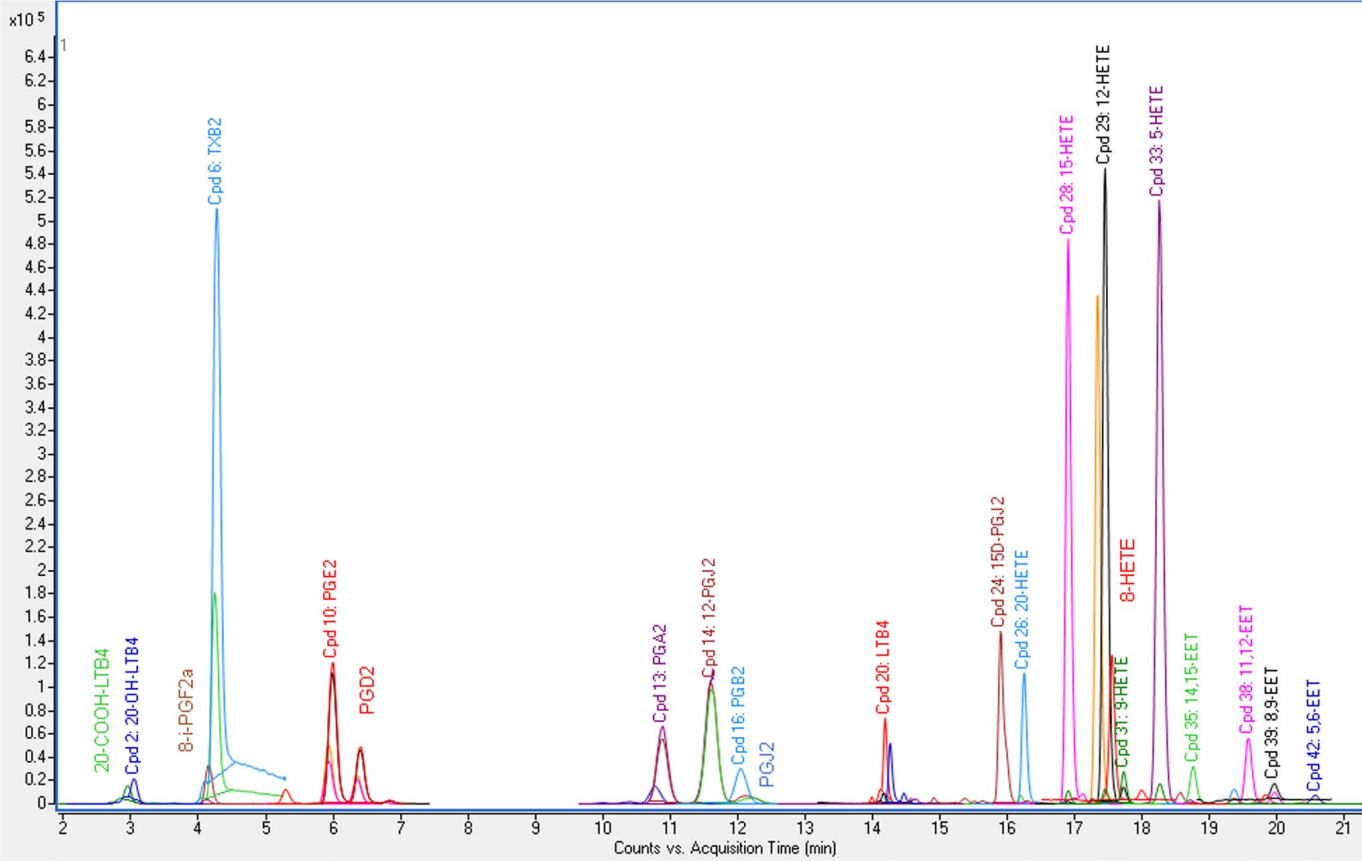
Overlaid chromatogram of the standard mixture of eicosanoids

As mentioned above, the structural similarity of the eicosanoids, particularly the isomers, requires excellent chromatographic separation. It is not sufficient to distinguish between eicosanoids that produced the same ion fragmentation pattern by using mass spectrometry alone. To obtain the optimal separation and sensitivity, the selection of chromatographic conditions was optimized for effective chromatographic resolution. Most lipid mediator-related studies employ C18-based columns to perform the separation [12, 13]. However, PGA_2_/PGJ_2_/Δ^12^-PGJ_2_ and 8,9-EET/11,12-EET were not resolved on the C18 columns, as the peak resolution *R* values were poor. Similarly, previous studies have failed to separate the mentioned isomers, which were quantified as mixtures [10, 14]. To resolve this problem, a phenyl-hexyl column was investigated. The column combines the productivity enhancements of superficially porous particle technology with phenyl-hexyl bonding, which delivers unique selectivity for compounds with aromatic groups, providing superior resolution. This column can also provide optimum separations of moderately polar compounds where typical C18 or C8 columns do not provide adequate resolution. PGA_2_/PGJ_2_/Δ^12^-PGJ_2_, a group of isomers that only differ in the position of olefinic bonds or functional groups, were perfectly separated on the phenyl-hexyl column. The separation of other critical compounds that shared the same MRM pairs, such as PGD_2_/PGE_2_ and 8,9-EET/11,12-EET, also exhibited adequate separations (Fig. 2). The overlaid chromatogram of the standard mixture of eicosanoids is shown in Fig. 3.

### Optimization of sample preparation

Several sample preparation strategies were evaluated, such as protein precipitation, liquid–liquid extraction (LLE) and SPE. The protein precipitation did not satisfy the quantisation limits of some low-concentration eicosanoids in biological samples. LLE showed the advantage of reducing the matrix effect and reasonable extraction efficiencies for EETs, HETEs and PGs but poor recovery for hydrophilic compounds such as leukotrienes. Overall, LLE with MTBE was not a sufficient sample preparation strategy. Then, an SPE method was investigated. HLB SPE is suitable for the extraction of a wide spectrum of compounds with different physicochemical properties. HLB SPE achieved acceptable recovery and precision and was subsequently used for our study. MeOH was chosen as the eluting solvent, given that ethyl acetate could cause the breakdown of cysteinyl leukotrienes according to a previous report [15].

### Method validation

**Fig. 4:**
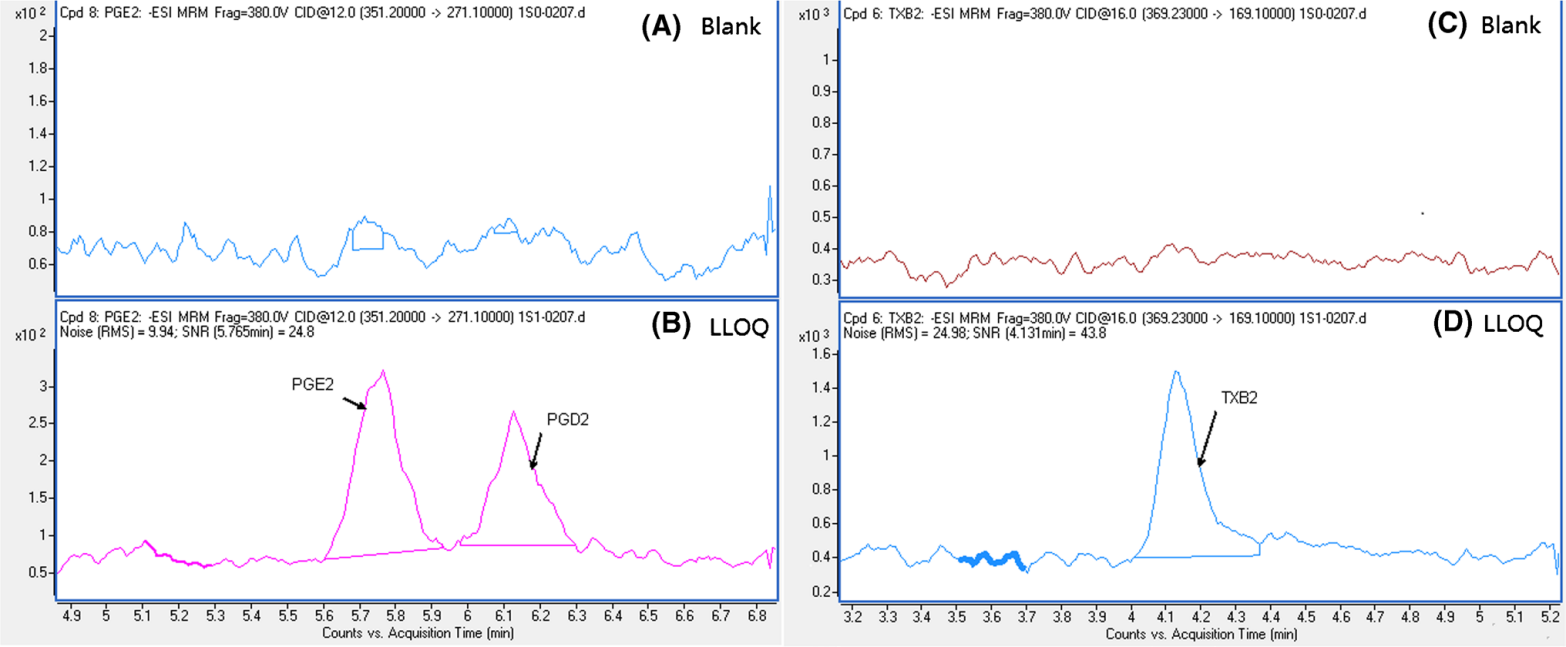
Chromatograms of diluted blank serum and LLOQ: examples of diluted blank serum and LLOQs of PGE2, PGD2 (**A**, **B**) and TXB2 (**C**, **D**)

Due to the lack of an analyte-free matrix, we prepared calibration curves in 10 × -diluted commercial double charcoal-stripped human serum. The linearity was generated by plotting the ratio of the analyte standard peak area to the internal standard peak area versus the amount of analyte standard using a weighting factor of 1/*x*. The standard calibration curves of the 25 compounds showed good linearity with calibration regression coefficients (*R*^2^) above 0.99. PGH_2_ yielded poor accuracy due to its instability. LTC_4_ obtained an unsatisfactory regression coefficient of less than 0.90. Dynamic ranges were used to cover the target eicosanoids at relevant physiological concentrations in the serum. The LLOQ and calibration curves of the 25 analytes are listed in Table S1. Several examples of the analysis of diluted blank serum and LLOQs are shown in Fig. 4. In diluted blank serum, no interference was observed for PGE_2_, PGD_2_ and TXB_2_ (Fig. 4A, C). The *S*/*N* of PGE_2_, PGD_2_ and TXB_2_ were greater than 10:1.

The precision and accuracy were examined by analysing the within-run and between-run variations in QC samples for each analyte (six replicates per concentration). Accuracy was calculated as the percentage of measured concentration relative to nominal concentration (RE%), whereas precision was determined as the coefficient of variation (CV%). The CV% of the within-run and the between-run precision were within 15%, and the within-run and the between-run accuracy ranged from 82.3 to 115.0% and 83.9 to 112.8%, respectively, indicating acceptable results for the determination of the analytes in human serum according to the criteria of FDA guidance. The results of precision and accuracy for analytes are listed in Table 2.

**Table 2:** Within-run and between-run precision and accuracy of eicosanoids

Compound name	Within-run precision (CV%)			Between-run precision (CV%)			Within-run accuracy (recovery%)			Between-run accuracy (recovery%)		
	L	M	H	L	M	H	L	M	H	L	M	H
20-COOH-LTB_4_	6.7	2.9	3.0	7.5	2.4	3.4	100.0	91.7	102.4	99.8	92.7	105.6
20-OH-LTB_4_	6.5	3.2	4.8	4.7	2.9	3.8	103.3	103.3	106.7	104.1	103.9	105.4
8-i-PGF_2a_	3.7	3.0	3.1	3.7	2.7	5.2	90.0	89.3	99.0	90.0	89.3	97.9
TXB_2_	5.1	1.9	1.6	4.2	2.0	1.1	99.0	106.0	101.3	101.2	106.4	101.6
PGE_2_	5.4	2.8	2.0	3.8	3.7	3.6	92.5	98.8	104.9	92.4	96.0	103.9
PGD_2_	2.7	3.3	3.7	3.9	5.2	3.9	92.5	99.5	95.6	93.9	95.1	96.9
PGA_2_	2.5	5.2	3.1	4.4	3.8	5.4	100.0	95.5	88.7	102.9	94.2	90.5
12-PGJ_2_	1.1	2.4	5.2	2.4	6.9	13.5	115.0	113.9	96.1	112.9	107.8	83.9
PGJ_2_	3.0	5.2	5.3	6.4	6.0	7.2	110.0	109.0	108.1	103.2	108.3	112.4
PGB_2_	8.2	3.7	1.1	4.9	4.8	1.9	101.7	109.0	102.3	101.4	103.2	102.5
LTD_4_	11.1	3.7	8.0	12.3	5.3	14.1	90.0	80.5	61.2	89.2	84.8	67.0
LTB_4_	5.0	4.0	5.1	4.1	7.7	7.0	100.0	82.3	86.8	100.0	91.3	93.8
LTE_4_	3.2	2.1	2.1	3.2	3.8	3.6	105.0	103.5	100.9	103.0	105.3	100.8
15D-PGJ_2_	2.9	5.0	3.6	4.2	4.5	2.3	113.3	106.0	103.3	109.3	107.9	104.4
20-HETE	3.2	5.8	2.4	3.5	5.2	3.0	103.3	115.0	108.4	104.9	109.7	109.3
15-HETE	5.2	2.1	3.8	3.7	2.8	2.7	91.5	96.4	97.8	93.8	94.7	99.1
12-HETE	2.4	1.5	2.4	1.8	1.2	2.2	114.0	104.4	103.0	104.8	107.9	104.7
8-HETE	2.4	5.1	4.0	2.7	5.1	5.0	103.8	98.5	92.2	101.6	98.1	97.1
9-HETE	2.4	3.8	2.4	2.8	4.2	3.6	102.5	92.8	93.7	101.7	95.6	97.3
5-HETE	1.1	2.8	4.6	1.6	2.0	3.5	108.1	107.9	108.0	108.5	111.8	105.4
14,15-EET	3.2	2.7	2.9	3.3	8.9	2.4	103.3	92.5	100.5	104.2	88.6	100.9
11,12-EET	3.1	4.5	4.9	3.8	9.8	3.8	106.7	95.3	96.7	104.6	84.6	95.0
5-OXO-EET	7.4	5.6	4.8	7.2	4.0	8.3	90.0	89.0	99.9	90.1	90.6	96.3
8,9-EET	3.8	1.6	1.7	3.0	1.7	2.2	98.8	104.8	102.7	100.0	103.5	101.0
5,6-EET	4.8	4.4	4.8	5.2	5.4	4.5	105.0	91.5	86.9	102.9	89.5	74.2

The extraction recovery of representative eicosanoids ranged from 64.5 to 136.8% (Table S2). The recoveries for leukotriene compounds (i.e. 20-COOH-LTB_4_, 20-OH-LTB_4_ and LTB_4_) had only moderate recoveries ranging from 64.5 to 69.8%. In contrast, prostaglandins, thromboxanes and hydroxy eicosanoids had excellent recoveries between 71.4 and 136.8%. The matrix effect ranged from 73.0 to 128.0%. The stability of LTB_4_ and EETs were supposed to be one of the reason for their low recoveries. Previous study [16] showed that both temperature and pH greatly affected the stability of LTB_4_ during SPE procedure and in vitro LTB_4_ was converted into 6-trans LTB_4_ and 6-trans-12-epi LTB_4_. On one hand, owing to the limited availability, some of the targets (such as 20-COOH-LTB_4_ and 20-OH-LTB_4_) were not calibrated by isotope-labelled analogues of their own. On the other hand, 20-COOH-LTB_4_ and 20-OH-LTB_4_ were considerably more polar than LTB_4_ due to the hydroxyl group or carboxyl group which may explain the poor recovery of leukotriene compounds in the biological samples.

The results of the stability experiment are summarized in Table S3 and Table S4. The analytes were stable in serum for 8 h within a 15% standard deviation except for PGD_2_, PGH_2_ and LTD_4_. PGD_2_, PGH_2_ and LTD_4_ were dramatically decreased in serum samples left for 8 h at room temperature. The extracted samples were stable at 4 °C in an autosampler for 24 h. For the freeze and thaw stability tests, QC samples were thawed at room temperature for 1 h and refrozen at − 80 °C for at least 12 h. This procedure was repeated twice, and the concentrations of samples subjected to freeze–thaw cycles were compared to those of freshly thawed samples. All eicosanoids were stable after a single freeze–thaw cycle, whereas LTD_4_, PGE_2_, PGD_2_, LTB_4_, 5-OXO-EET and 5,6-EET showed significant loss after three freeze–thaw cycles.

### Analyses of eicosanoids and their metabolites in biological samples

To answer the question of whether differences were observed between patients with and without H1N1 virus infection, the validated UPLC coupled to tandem mass spectrometry method was applied to determine the target eicosanoids in 74 serum samples, using a volume of 200 μL per sample. Samples were obtained from healthy individuals and patients with severe pneumonia induced by influenza virus or bacteria. The clinical characteristics of the study population are summarized in Table S5. All subjects were aged 19 ~ 96 years. More males (*n* = 50) than females (*n* = 24) participated. Concentrations of 25 metabolites from 74 serum samples were quantified (Table 3). The concentrations of eicosanoids in serum obtained from the present study are in agreement with those found previously published by others [17–21]. For example, the concentrations of PGs (PGE_2_, PGD_2_ and PGA_2_) were 0.39, 0.05 and 0.13 ng/mL in the present study and 0.43, 0.39 and 0.10 ng/mL in Wang et al. [21], and the concentrations of EETs (5,6-EET, 8,9-EET, 11,12-EET and 14,15-EET) in our study were 0.06, 0.03, 0.13 and 0.11 ng/mL and 0.33, 0.43, 0.17 and 0.35 ng/mL in Gouveia-Figueira et al. [17], respectively. Compared to other eicosanoids, concentration levels of 12-HETE and TXB_2_ in serum in our study are high as their basic expressions in human serum were high [18, 20], which were similar to previous studies. Besides, the activation of platelet-derived 12-LOX in serum during clotting may result in a higher level of 12-HETE [22].

**Table 3:** Comparison of eicosanoids and their metabolite concentrations quantified in 74 serum samples

Metabolite	Health (ng/mL)		Severe influenza pneumonia (ng/mL)		Severe bacterial pneumonia (ng/mL)	
	Mean	SD	Mean	SD	Mean	SD
20-COOH-LTB4	0.28	0.57	0.44	0.55	0.34	0.74
20-OH-LTB4	0.18	0.25	0.32	0.35	0.10	0.18
8-i-PGF2a	0.09	0.06	0.09	0.15	0.09	0.09
TXB2	42.36	29.80	15.28**	22.10	23.59^#^	29.81
PGE2	0.39	0.30	0.30	0.47	0.80	1.53
PGD2	0.05	0.05	0.09	0.16	0.88	2.77
PGA2	0.13	0.09	0.13	0.17	0.19	0.36
12-PGJ2	0.07	0.05	0.07	0.14	0.07	0.08
PGB2	0.24	0.19	0.22	0.36	0.20	0.17
PGJ2	0.30	0.23	0.23	0.29	0.18	0.16
LTB4	0.77	1.04	0.11*	0.16	0.30	0.32
LTE4	0.23	0.23	0.13	0.11	0.07^##^	0.08
15D-PGJ2	0.04	0.03	0.07	0.15	0.03	0.02
20-HETE	0.13	0.05	0.21*	0.20	0.19	0.14
15-HETE	4.47	2.75	3.45	4.37	5.67	6.15
12-HETE	307.43	199.14	198.03	204.81	326.67	270.91
8-HETE	0.99	0.76	1.08	1.02	2.87^#^	4.62
9-HETE	1.87	4.17	1.66	3.49	4.20	9.86
5-HETE	2.64	1.79	2.25	2.74	30.34^#^	77.44
14,15-EET	0.06	0.02	0.12*	0.16	0.06	0.02
11,12-EET	0.03	0.01	0.07**	0.10	0.04	0.01
5-OXO-EET	0.41	0.44	0.22	0.22	1.77	5.62
8,9-EET	0.13	0.16	0.42	0.97	0.68	2.45
5,6-EET	0.11	0.05	0.18	0.13	0.15	0.13

^*^Shown is the *P* value of the influenza pneumonia group compared to the healthy group (^*^P < 0.05, ^**^P < 0.01, ^***^P < 0.001).

^#^Shown is the *P* value of the bacterial pneumonia group compared to the healthy group (^#^*P* < 0.05, ^##^*P* < 0.01, ^###^*P* < 0.001).

**Fig. 5:**
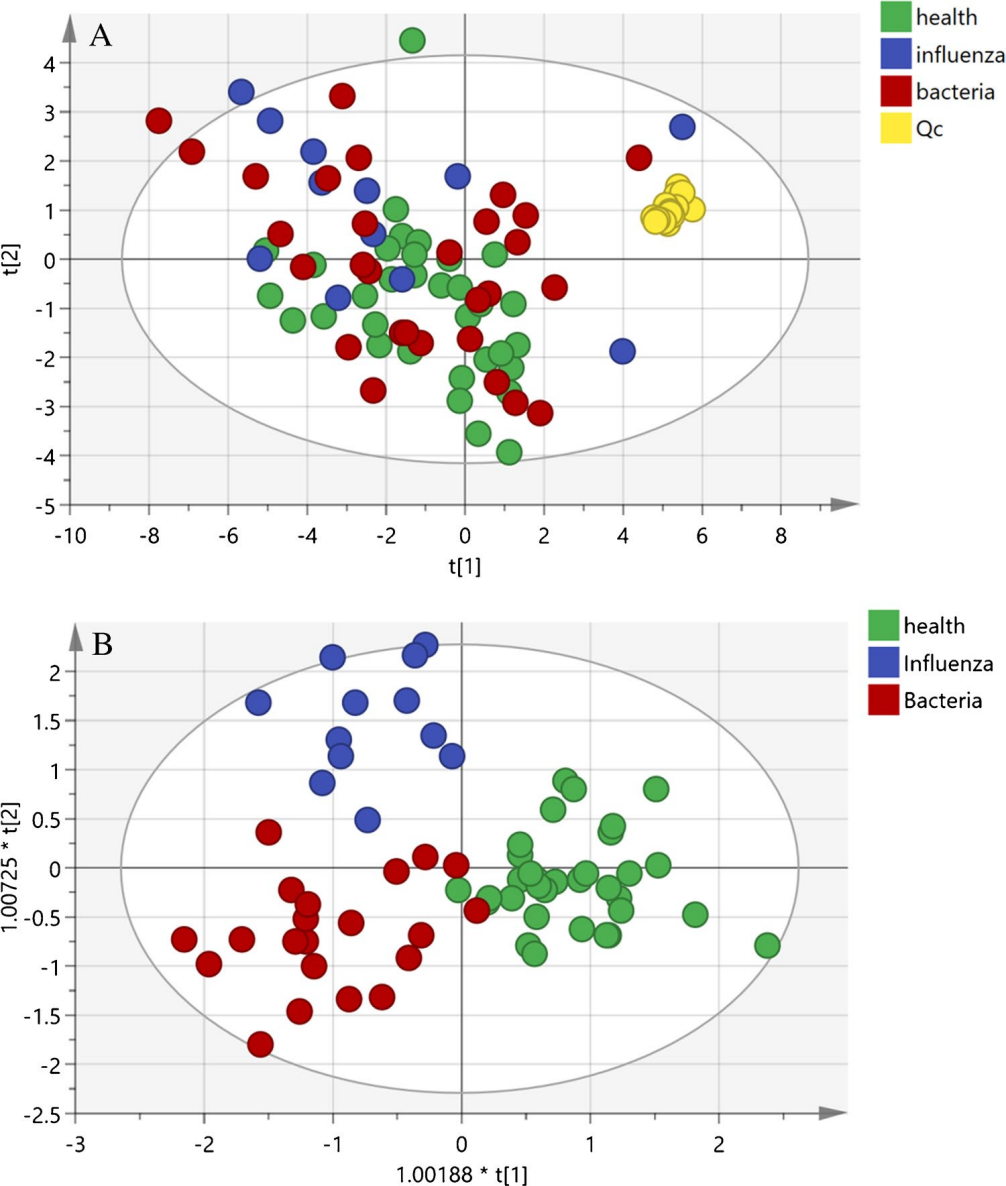
**A** PCA score plots of 74 serum samples and 18 QC samples. **B** OPLS-DA performed on the data of serum samples from healthy individuals and patients diagnosed with severe pneumonia induced by influenza or by bacteria. (*R*^2^*X* = 0.736, *R*^2^*Y* = 0.724, *Q*.^2^ = 0.518)

Host synthesis of bioactive lipids is in part specific for certain pathogens [23]. However, the differences of eicosanoids in serum between severe influenza pneumonia and severe bacterial pneumonia remain unclear. Finding out the difference helps to make a better and more precise diagnosis on the cause of pneumonia. Hence, we first established an unsupervised study with principal component analysis (PCA) on all the samples, including 18 QC samples. QC samples clustered together in the PCA score plot, indicating the good stability and reproducibility of the LC–MS method applied to biological samples (Fig. 5A). Orthogonal partial least square discriminant analysis (OPLS-DA) was carried out to analyse the differences among the healthy group, severe influenza pneumonia group and severe bacterial pneumonia group. OPLS-DA provided perfect discrimination among the three groups (Fig. 5B). Compared to healthy individuals, significant differences in several metabolites were obtained. Concentrations of 20-HETE, 14,15-EET and 11,12-EET increased, while that of LTB4 was reduced in influenza severe pneumonia patients. Severe influenza infection may have occurred because of the low level of LTB_4_. Pernet et al. demonstrated that compared to control mice, LTB_4_-deficient mice are more susceptible to IAV infection [24]. Similar to our study, Schultz et al. showed that the concentration of 20-HETE in mice lung increased one day after influenza infection and showed no significant difference in bacteria-infected mice. Besides, 11,12-EET and 14,15 EET were also found increased 2 days after influenza infection but still no difference in the bacteria-infected mice [23].

**Fig. 6:**
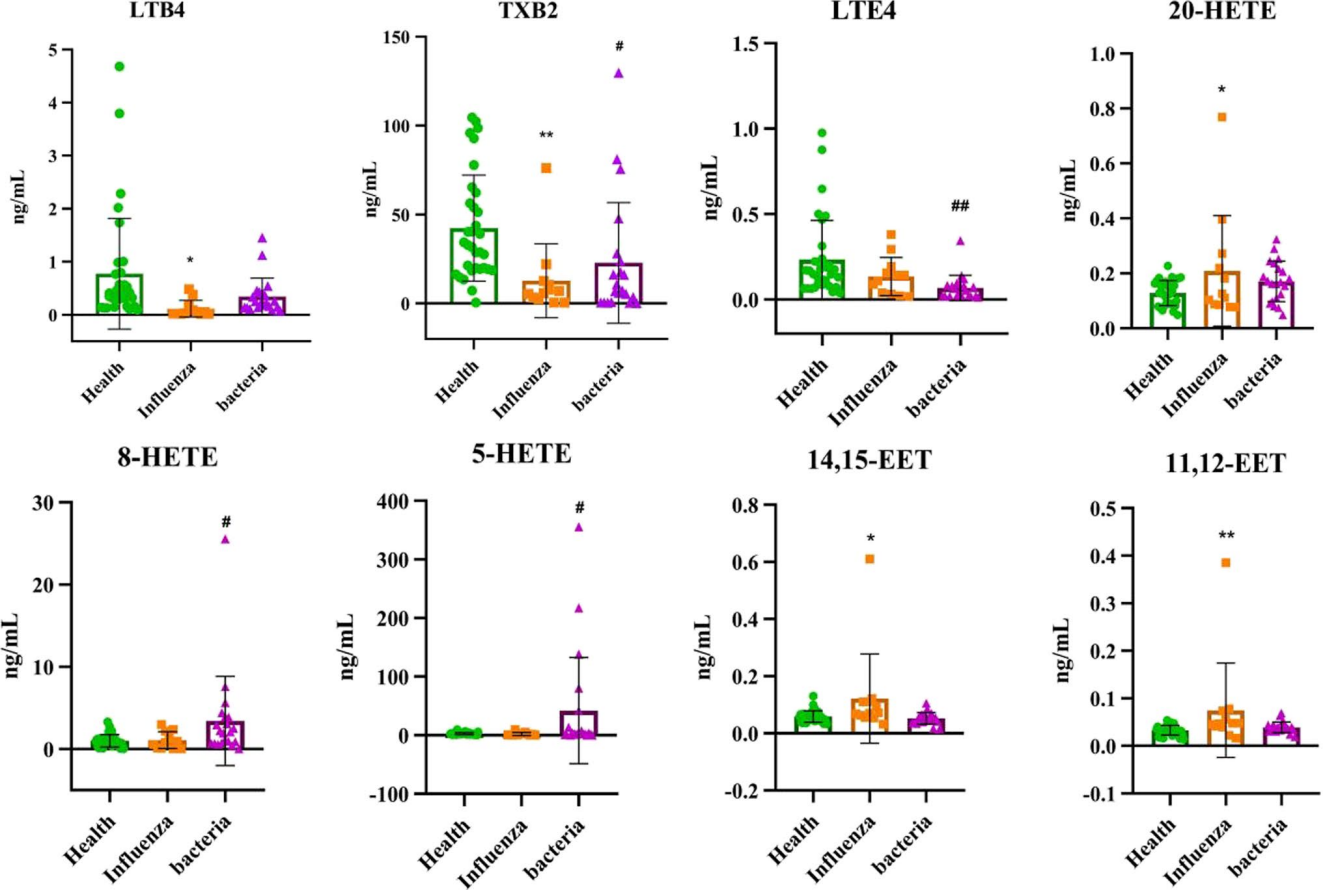
Scatter plots of eight significantly altered metabolites in serum samples of the healthy group, severe influenza pneumonia group and severe bacterial pneumonia group. ^*^Shown is the *P* value of the influenza pneumonia group compared to the healthy group (^*^P < 0.05, ^**^P < 0.01, ^***^P < 0.001). ^#^Shown is the *P* value of the bacterial pneumonia group compared to the healthy group (^#^*P* < 0.05, ^##^*P* < 0.01, ^###^*P* < 0.001)

Moreover, the concentrations of 8-HETE and 5-HETE were upregulated, while that of LTE_4_ was downregulated in severe bacterial pneumonia patients. Among all the metabolites, TXB_2_ was downregulated in severe pneumonia patients induced by influenza or bacteria and was reduced in the former group (Table 3 and Fig. 6). A previous report also demonstrated that LoxA secreted by *Pseudomonas aeruginosa* plays an important role in the increasing of 8-HETE and 5-HETE in BALF, human lung epithelial NCI-H292 cells and human blood neutrophils (25).

## Conclusion

A targeted UPLC–MS/MS method to simultaneously quantify 25 eicosanoids in human serum was developed and validated. The results presented here describe a robust, reliable and sensitive assay for the detection of metabolites derived from AA. To ensure the detection sensitivity and coverage of major targets, we carefully optimized the LC–MS/MS conditions. The coverage of analytes needs to be balanced against the sensitivity. This study focuses on improving the resolution to distinguish isomeric and isobaric compounds using a phenyl-hexyl column. In addition, we employed the dMRM mode and a polarity switching procedure to guarantee sensitivity. This method was applied to profile the eicosanoids in severe pneumonia patients with different sources of infection, and our finding that different characteristic metabolite profiles may help discriminate the induction of severe pneumonia patients. Further study will be carefully evaluated after larger prospective cohorts in the future.

## Supplementary Information

Below is the link to the electronic supplementary material.

Supplementary file1 (DOCX 39 KB)
