# Supplementary material for: Simultaneous profiling and quantification of 25 eicosanoids in human serum by ultrahigh-performance liquid chromatography coupled to tandem mass spectrometry
Source: Anal Bioanal Chem. 2022 Nov 8;414(29-30):8233–44. doi: 10.1007/s00216-022-04351-6 (PMC9712357; doi:10.1007/s00216-022-04351-6)
Supplement: Supplementary file 1 — Supplementary file1 (DOCX 39 KB) [file 216_2022_4351_MOESM1_ESM.docx]

**Supplementary data**

**Table S1.** The internal standard (IS), linearity range, correlation coefficient, and LLOQ for eicosanoids.

| Compound Name | IS | Linear Range（ng/mL） | correlation coefficient(R^2^) | LLOQ（ng/mL） |
| --- | --- | --- | --- | --- |
| 20-COOH-LTB_4_ | 8-i-PGF_2a_-d4 | 0.048~50.0 | 0.9987 | 0.048 |
| 20-OH-LTB_4_ | 8-i-PGF_2a_-d4 | 0.048~50.0 | 0.9976 | 0.048 |
| 8-i-PGF_2a_ | 8-i-PGF_2a_-d4 | 0.048~100.0 | 0.9986 | 0.048 |
| TXB_2_ | TXB_2_-d4 | 0.24~500.0 | 0.9981 | 0.24 |
| PGE_2_ | PGE_2_-d4 | 0.048~100.0 | 0.9988 | 0.048 |
| PGD_2_ | PGD_2_-d4 | 0.048~100.0 | 0.9980 | 0.048 |
| PGA_2_ | PGA_2_-d4 | 0.048~100.0 | 0.9948 | 0.048 |
| 12-PGJ_2_ | PGA_2_-d4 | 0.048~100.0 | 0.9974 | 0.048 |
| PGB_2_ | PGA_2_-d4 | 0.048~100.0 | 0.9989 | 0.048 |
| PGJ_2_ | PGA_2_-d4 | 0.098~100.0 | 0.9924 | 0.098 |
| LTD_4_ | LTB_4_-d4 | 0.098~100.0 | 0.9909 | 0.098 |
| LTE_4_ | LTE_4_-d5 | 0.048~100.0 | 0.9919 | 0.048 |
| LTB_4_ | 20-HETE-d6 | 0.195~100.0 | 0.9967 | 0.195 |
| 15D-PGJ_2_ | LTE_4_-d5 | 0.048~100.0 | 0.9918 | 0.048 |
| 20-HETE | 20-HETE-d6 | 0.048~100.0 | 0.9974 | 0.048 |
| 15-HETE | 12-HETE-d8 | 0.24~500.0 | 0.9991 | 0.24 |
| 12-HETE | 12-HETE-d8 | 0.88~900.0 | 0.9983 | 0.88 |
| 8-HETE | 20-HETE-d6 | 0.048~100.0 | 0.9953 | 0.048 |
| 9-HETE | 20-HETE-d6 | 0.098~100.0 | 0.9964 | 0.098 |
| 5-HETE | 14,15-EET-d11 | 0.44~900.0 | 0.9991 | 0.44 |
| 14,15-EET | 5-HETE-d8 | 0.048~50.0 | 0.9988 | 0.048 |
| 11,12-EET | 11,12-EET-d11 | 0.048~50.0 | 0.9985 | 0.048 |
| 5-OXO-EET | 8,9-EET-d11 | 0.048~100.0 | 0.9976 | 0.048 |
| 8,9-EET | 8,9-EET-d11 | 0.195~100.0 | 0.9972 | 0.195 |
| 5,6-EET | 5,6-EET-d11 | 0.048~50.0 | 0.9962 | 0.048 |

**Table S2** The recovery and normalized absolute matrix effect of eicosanoids

| Compound Name | RE（%）± SD | | ME (%) ± SD | |
| --- | --- | --- | --- | --- |
|  | L | H | L | H |
| 20-COOH-LTB_4_ | 75.3±7.2 | 94.0± 2.4 | 108.4±2.6 | 91.1±4.3 |
| 20-OH-LTB_4_ | 69.4±3.5 | 90.8±2.0 | 107.4±6.0 | 92.3±5.3 |
| 8-i-PGF_2a_ | 99.5±5.9 | 102.2±2.8 | 104.9±4.5 | 92.9±5.2 |
| TXB_2_ | 96.4±2.3 | 96.9±1.3 | 99.9±4.1 | 94.6±4.6 |
| PGE_2_ | 100.0±3.2 | 102.0±2.5 | 100.0±3.4 | 92.8±5.2 |
| PGD_2_ | 99.1±3.0 | 100.7±2.7 | 97.0±3.1 | 93.2±5.1 |
| PGH_2_ | 124.3±5.5 | 99.5±2.3 | 82.8±39.0 | 92.0±5.5 |
| PGA_2_ | 110.8±7.3 | 101.1±3.5 | 96.0±4.2 | 92.5±6.1 |
| 12-PGJ_2_ | 103.3±11.6 | 93.6±2.8 | 106.5±4.1 | 89.4±5.0 |
| PGB_2_ | 100.0±10.6 | 99.8±3.2 | 104.3±6.7 | 92.1±5.7 |
| PGJ_2_ | 98.2±2.5 | 95.1±3.0 | 91.7±3.0 | 91.4±5.4 |
| LTC_4_ | 90.3±6.0 | 96.9±3.4 | 93.3±4.7 | 111.8±6.9 |
| LTB_4_ | 72.8±8.3 | 86.5± 3.3 | 86.8±4.6 | 87.4±4.7 |
| LTE_4_ | 88.3±9.2 | 93.7±6.5 | 107.1±4.7 | 116.1±11.9 |
| LTD_4_ | 93.0±7.8 | 88.4±9.3 | 97.3±3.7 | 98.7±16.6 |
| 15D-PGJ_2_ | 87.6±3.1 | 81.8±5.2 | 104.0±4.0 | 78.8±5.8 |
| 20-HETE | 100.0±4.3 | 92.6±3.9 | 92.9±2.5 | 84.6±5.3 |
| 15-HETE | 81.6±6.1 | 84.4±2.7 | 115.3±3.9 | 87.3±5.7 |
| 12-HETE | 86.8±4.5 | 83.9±2.2 | 110.2±4.2 | 86.8±5.1 |
| 8-HETE | 90.1±11.8 | 85.7±3.0 | 103.4±9.1 | 85.4±5.6 |
| 9-HETE | 114.8±22.0 | 85.8±1.3 | 108.8±11.4 | 88.9±4.4 |
| 5-HETE | 86.8±7.0 | 85.8±3.4 | 111.6±5.8 | 91.7±6.5 |
| 14,15-EET | 76.8±7.0 | 74.5±2.1 | 110.1±4.9 | 86.3±5.6 |
| 11,12-EET | 82.3±5.1 | 75.0±3.3 | 105.8±3.7 | 89.8±6.7 |
| 5-OXO-EET | 85.9±9.3 | 76.0±4.2 | 95.3±3.6 | 91.4±6.4 |
| 8,9-EET | 87.3±11.2 | 74.0±2.6 | 90.1±3.7 | 89.0±6.9 |
| 5,6-EET | 79.3±5.8 | 69.7±2.7 | 93.3±3.0 | 84.5±4.4 |

**Table S3** The post-preparative stability of eicosanoids

| Compound Name | post-preparative stability 8 h, % | | | 14 h, % | | | 24 h, % | | |
| --- | --- | --- | --- | --- | --- | --- | --- | --- | --- |
|  | L | M | H | L | M | H | L | M | H |
| 20-COOH-LTB_4_ | 95.4 | 95.0 | 97.2 | 96.2 | 96.3 | 97.0 | 92.7 | 98.7 | 94.4 |
| 20-OH-LTB_4_ | 98.7 | 95.2 | 99.3 | 98.7 | 97.6 | 98.6 | 105.4 | 100.1 | 99.6 |
| 8-i-PGF_2a_ | 104.4 | 95.4 | 104.1 | 105.6 | 98.8 | 102.9 | 108.7 | 99.5 | 106.0 |
| TXB_2_ | 103.4 | 101.8 | 100.4 | 99.3 | 102.4 | 100.3 | 105.0 | 107.3 | 99.3 |
| PGE_2_ | 100.0 | 100.0 | 98.5 | 100.2 | 106.0 | 98.9 | 99.0 | 96.8 | 97.5 |
| PGD_2_ | 99.7 | 96.2 | 100.7 | 98.3 | 90.9 | 93.3 | 92.5 | 81.7 | 97.1 |
| PGA_2_ | 95.8 | 96.4 | 95.1 | 95.6 | 94.6 | 93.9 | 89.0 | 92.9 | 92.1 |
| 12-PGJ_2_ | 106.5 | 106.8 | 101.1 | 102.1 | 104.5 | 104.7 | 96.9 | 104.1 | 103.5 |
| PGB_2_ | 107.1 | 101.3 | 98.7 | 102.9 | 98.3 | 97.2 | 104.5 | 99.3 | 100.0 |
| PGJ_2_ | 99.1 | 98.4 | 99.6 | 95.4 | 98.6 | 91.8 | 102.0 | 103.0 | 100.2 |
| LTD_4_ | 101.6 | 93.8 | 99.3 | 98.0 | 92.2 | 103.4 | 84.7 | 89.7 | 92.8 |
| LTB_4_ | 93.4 | 89.2 | 89.4 | 96.0 | 103.9 | 102.0 | 82.9 | 92.5 | 89.9 |
| LTE_4_ | 97.4 | 100.6 | 102.1 | 96.0 | 96.6 | 99.1 | 91.7 | 98.0 | 100.4 |
| 15D-PGJ_2_ | 94.4 | 100.6 | 100.4 | 97.9 | 104.3 | 101.5 | 96.9 | 101.1 | 100.6 |
| 20-HETE | 101.5 | 97.4 | 99.3 | 93.2 | 97.2 | 95.1 | 99.7 | 98.0 | 97.0 |
| 15-HETE | 102.0 | 98.8 | 99.3 | 100.6 | 101.0 | 100.1 | 99.0 | 98.6 | 101.0 |
| 12-HETE | 103.6 | 100.1 | 100.0 | 103.1 | 98.9 | 100.0 | 100.0 | 96.7 | 99.1 |
| 8-HETE | 102.8 | 98.7 | 96.8 | 101.9 | 96.6 | 97.0 | 101.5 | 96.2 | 97.3 |
| 9-HETE | 98.0 | 92.1 | 104.1 | 97.4 | 101.0 | 101.8 | 97.9 | 103.5 | 103.8 |
| 5-HETE | 101.0 | 100.4 | 100.6 | 97.5 | 101.2 | 99.6 | 99.0 | 99.8 | 99.0 |
| 14,15-EET | 106.2 | 103.4 | 100.6 | 103.3 | 105.2 | 101.3 | 99.6 | 103.3 | 101.1 |
| 11,12-EET | 102.6 | 101.2 | 97.9 | 98.3 | 103.0 | 95.6 | 96.9 | 101.4 | 95.2 |
| 5-OXO-EET | 106.4 | 104.7 | 99.2 | 103.4 | 98.9 | 99.3 | 96.6 | 108.4 | 99.8 |
| 8,9-EET | 100.6 | 104.4 | 98.7 | 101.1 | 98.3 | 99.1 | 98.9 | 101.7 | 99.9 |
| 5,6-EET | 110.1 | 97.1 | 108.6 | 101.2 | 106.9 | 106.2 | 96.9 | 87.2 | 100.8 |

**Table S4** The stability of eicosanoids under various storage conditions

| Compound Name | RT 8 h,% | | | Freeze and thaw ,%,  Cycle 1 | | | Freeze and thaw , %,  Cycle 2 | | |
| --- | --- | --- | --- | --- | --- | --- | --- | --- | --- |
|  | L | M | H | L | M | H | L | M | H |
| 20-COOH-LTB_4_ | 78.7 | 91.3 | 94.3 | 94.1 | 93.7 | 96.2 | 87.5 | 91.1 | 94.9 |
| 20-OH-LTB_4_ | 88.7 | 93.8 | 99.8 | 105.1 | 93.9 | 100.1 | 97.4 | 95.0 | 98.7 |
| 8-i-PGF_2a_ | 93.2 | 95.6 | 93.9 | 97.2 | 93.7 | 97.5 | 90.7 | 99.1 | 96.2 |
| TXB_2_ | 92.5 | 94.3 | 98.3 | 99.3 | 103.6 | 99.0 | 93.8 | 96.0 | 100.4 |
| PGE_2_ | 78.3 | 98.7 | 85.7 | 98.2 | 93.3 | 92.5 | 72.7 | 83.6 | 87.3 |
| PGD_2_ | 42.1 | 41.1 | 57.4 | 93.6 | 88.4 | 100.9 | 41.6 | 43.6 | 72.6 |
| PGA_2_ | 100.4 | 82.2 | 83.5 | 91.7 | 90.4 | 92.3 | 92.8 | 86.7 | 83.0 |
| 12-PGJ_2_ | 101.3 | 105.7 | 104.9 | 99.1 | 104.7 | 86.0 | 99.4 | 110.0 | 94.4 |
| PGB_2_ | 104.0 | 95.7 | 93.4 | 102.2 | 98.3 | 98.3 | 93.8 | 97.7 | 98.7 |
| PGJ_2_ | 110.1 | 107.6 | 99.2 | 96.3 | 92.0 | 97.0 | 108.4 | 112.9 | 101.1 |
| LTD_4_ | 75.1 | 66.7 | 59.4 | 97.2 | 80.8 | 89.2 | 83.1 | 57.6 | 50.8 |
| LTB_4_ | 92.4 | 104.0 | 87.8 | 68.6 | 100.4 | 94.6 | 66.6 | 91.4 | 92.9 |
| LTE_4_ | 99.6 | 93.9 | 101.2 | 90.7 | 100.3 | 102.6 | 104.4 | 96.6 | 101.5 |
| 15D-PGJ_2_ | 98.3 | 111.4 | 97.9 | 92.7 | 89.9 | 97.4 | 85.5 | 92.5 | 93.1 |
| 20-HETE | 85.7 | 87.3 | 100.5 | 97.4 | 94.4 | 93.7 | 90.2 | 97.0 | 92.5 |
| 15-HETE | 99.3 | 98.9 | 99.9 | 98.2 | 99.4 | 99.6 | 88.8 | 98.7 | 100.1 |
| 12-HETE | 100.5 | 93.3 | 97.5 | 111.3 | 96.9 | 98.9 | 103.4 | 99.9 | 97.1 |
| 8-HETE | 102.5 | 91.2 | 87.4 | 100.1 | 93.7 | 98.1 | 98.9 | 103.8 | 99.2 |
| 9-HETE | 97.5 | 90.5 | 100.8 | 96.9 | 105.0 | 104.0 | 96.0 | 100.1 | 103.8 |
| 5-HETE | 94.3 | 97.2 | 96.6 | 102.0 | 97.8 | 97.0 | 92.3 | 98.0 | 94.9 |
| 14,15-EET | 91.7 | 95.3 | 95.1 | 105.9 | 101.5 | 96.5 | 87.7 | 94.3 | 96.6 |
| 11,12-EET | 89.8 | 94.0 | 96.5 | 98.9 | 96.7 | 93.7 | 91.6 | 97.7 | 97.9 |
| 5-OXO-EET | 93.2 | 87.7 | 90.4 | 103.4 | 104.6 | 99.6 | 79.3 | 83.4 | 88.7 |
| 8,9-EET | 102.9 | 98.3 | 97.1 | 101.9 | 98.7 | 98.3 | 98.4 | 98.4 | 96.8 |
| 5,6-EET | 102.2 | 86.1 | 95.1 | 106.3 | 91.1 | 108.4 | 62.1 | 55.8 | 69.2 |

**Table S5** Clinical characteristics of the study groups

| Clinical characteristic | Health  (n=34) | Severe Influenza pneumonia patients  (n=12) | Severe Bacterial Pneumonia patients  (n=28) |
| --- | --- | --- | --- |
| Age | 53(32-78) | 49.5(32-87) | 67(19-96) |
| Sex: male(female) | 23(11) | 7(5) | 20(8) |
| Non-survivors | / | 0 | 2 |
| PaO2/FiO2 | / | 226.8(42.5-350.9) | 326.3(123.7-769.1) |

Values are medians (min to max), or n.
